# Autoimmune Encephalitis-like Presentation of Glioblastoma: Get to Know This Rare Occurrence

**DOI:** 10.3390/jcm14113807

**Published:** 2025-05-29

**Authors:** Alberto Negro, Vincenzo D’Agostino, Eugenio Maria Covelli, Laura Gemini, Eduardo Gragnano, Mario Tortora, Andrea Elefante, Luisa Chiapparini, Camilla Russo

**Affiliations:** 1 Neuroradiology Unit, Ospedale del Mare, via Enrico Russo, 80147 Naples, Italy; alberto.negro@hotmail.it (A.N.); dagovin79@gmail.com (V.D.); laura.gemini93@gmail.com (L.G.); 2 Neuroradiology Unit, “Sant’Anna e San Sebastiano” Hospital, 81100 Caserta, Italy; eugenio.covelli@gmail.com; 3 Neuroradiology Unit, Santobono-Pausilipon Children’s Hospital, 80129 Naples, Italy; 4 Department of Advanced Biomedical Sciences, University of Naples “Federico II”, 80131 Naples, Italy; eduardogragnano@gmail.com (E.G.); mario.tortora@ymail.com (M.T.); aelefant@unina.it (A.E.); 5 Neuroradiology Department, Fondazione IRCCS Policlinico S. Matteo, 27100 Pavia, Italy; l.chiapparini@smatteo.pv.it

**Keywords:** glioblastoma, autoimmune encephalitis, differential diagnosis, magnetic resonance imaging

## Abstract

**Background**: Glioblastoma (GBM) is the most common primary brain tumor in adults, with a poor prognosis and survival. Although typically presenting with focal neurological deficits, seizures, or cognitive decline, GBM can occasionally mimic autoimmune encephalitis (AE), leading to significant diagnostic delay. The overlap in clinical, radiological, and serological findings between GBM and AE underscores the need for thorough evaluation. **Methods**: We retrospectively reviewed cases of patients diagnosed between 2016 and 2023 with pathology-confirmed GBM, critically rethinking those cases initially diagnosed with AE at symptom onset. The diagnostic workup included magnetic resonance imaging (MRI), cerebrospinal fluid (CSF) analysis, autoantibody testing, and whole-body nuclear scanning to exclude extracranial malignancies. **Results**: We found five female patients diagnosed with GBM who initially presented with signs and symptoms suggestive for AE. Initial MRI showed non-specific brain tissue alterations, without definitive tumor features. CSF analysis was largely unremarkable, though some cases exhibited positive autoantibodies. Despite therapy, clinical deterioration and follow-up MRI revealed infiltrative intra-axial lesions with contrast enhancement, leading to pathology-confirmed GBM diagnoses. All patients had poor prognoses, with a mean survival of 10 ± 4 months. **Conclusions**: GBM can mimic AE, delaying appropriate treatment. In patients with atypical MRI findings and suboptimal response to therapy, early follow-up imaging and biopsy should be considered to exclude malignancy. A multidisciplinary approach is critical for timely diagnosis and improved management.

## 1. Introduction

Among primary brain neoplasms, the most frequent lesion type of adulthood is represented by glioblastoma (GBM), globally accounting for 50% of all gliomas, with a peak incidence in patients aged between 55 and 65 years and a slightly higher prevalence within the male population [1]; this tumor is characterized by high local biological aggressiveness, a low tendency for dissemination outside the central nervous system (CNS), and overall poor prognosis with short survival (survival rate < 5% at 5 y after the initial diagnosis; median survival of 1.5 y from initial diagnosis) [2]. The vast majority of GBMs originate in the elderly with no clinical or radiological evidence of precursor lesions (about 90–95% cases, also known as primary or de novo GBMs), while only a minority of GBM cases derived from the malignant transformation of pre-existing low-grade gliomas and usually affected younger patients (about 5–10% cases, also known as secondary GBMs) [3]. Regardless of primary or secondary, GBMs are almost indistinguishable at pathology and may share very similar clinical–radiological features at diagnosis.

From a radiological standpoint, despite the first-level examination to rule out the presence of brain neoplasm, usually represented by computed tomography (CT), the golden standard for GBM imaging characterization is represented by magnetic resonance imaging (MRI), widely used for both diagnostic and treatment monitoring purposes. At diagnosis, conventional MRI typically reveals the presence of a single expansive lesion within cerebral hemispheres (frontal lobe, deep white matter, corpus callosum, thalamus, and basal nuclei—more uncommon localizations such as the cerebellum and spinal cord are only occasionally observed), with an irregular peripheral solid enhancement surrounding a central necrotic core, associated with hemorrhagic components (when present) and an extensive halo of peri-tumoral edema containing neoplastic cellular infiltrates [4,5]. Uncommon presentations include evidence of multiple enhancing lesions, named multifocal (when different enhancing foci are connected by the same edema halo) and multicentric (when no connection between different enhancing foci is visible at MRI) [6]. Coupled to conventional MRI, advanced MRI techniques are widely adopted to provide additional information and further characterize GBMs, encompassing perfusion-weighted imaging (PWI) techniques, mainly including dynamic susceptibility contrast (DSC) and dynamic contrast enhancement (DCE); functional MRI (fMRI) and diffusion tensor imaging (DTI), mainly used to identify eloquent cortical areas and major white matter tracts in order to define focal infiltration and minimize postsurgical deficits; and MR spectroscopy (MRS) to define metabolic fingerprinting and assess infiltrating neoplastic edema beyond the margin of solid contrast enhancement in the first GBM diagnosis, but also to early-identify metabolic abnormalities in post-operative GBM margins in order to predate the onset of contrast enhancement indicative of tumor recurrence [7].

From a clinical standpoint, early symptoms suggestive for the presence of GBM are polymorphic and non-specific, largely depending on tumor dimension and location in the brain. In the case of larger lesions and for tumors causing hydrocephalus, early symptoms may include headache, vomiting, or visual disturbances due to the rise in intracranial pressure; conversely, in the case of smaller tumors, symptoms are more variable and insidious ranging from headache, personality changes or cognitive deterioration, focal or generalized seizures in otherwise non-epileptic patients, motor weakness, speech disturbances, vision or hearing impairment, and other progressive focal neurological deficits due to the localization of the lesion in eloquent anatomical regions within the CNS. Occasionally, GBM may also present with encephalitic symptoms, with unclear clinic–radiological findings frequently resulting in significant diagnostic delay and overall poor prognosis; indeed, some reports called GBM onset into question as a potential encephalitis mimic (both as autoimmune-like and infectious-like presentation) [8,9,10], whereas few other authors suggested the co-occurrence of glial tumors and encephalitis [11,12]. In particular, autoimmune encephalitis (AE), which either precedes or accompanies GBM, is a rare and complex phenomenon in which the tumor may trigger an aberrant immune response against neural antigens, leading to inflammatory involvement of the brain parenchyma, often complicating early recognition and management of the underlying malignancy. Despite isolated attempts [9], the actual correlation between GBM and encephalitic phenomena at MRI as well as their potential association remains uncharted territory. In this case series, we retrospectively collected and revised clinical–radiological evidence of patients with a final diagnosis of GBM who originally presented with the atypical and misleading features of encephalitis-like onset, speculating on the possible connection between glial neoplasm and immune-mediated neurologic phenomena.

## 2. Materials and Methods

We retrospectively reviewed a series of patients collected between 2016 and 2023 with a pathological diagnosis of GBM according to 2016 WHO criteria and their 2021 WHO update, who were first diagnosed as a possible AE and who were only later discovered with glial neoplasm at longitudinal follow-up. We revised demographical, clinical, laboratory, and imaging findings of these patients both at diagnosis and follow-up; patients with unavailable data and absent or incomplete multidimensional examination at diagnosis were excluded. In addition, patients with intellectual disability, previous neuropsychiatric disorders, a past history of brain neoplasm, and/or more complex neurological presentations (i.e., encephalomyelitis) were not included, in order to avoid possible confounders.

All the identified patients at clinical onset underwent unenhanced CT and resting-state electroencephalography (EEG); due to the persistence of clinical symptoms despite negative CT, contrast-enhanced MRI examination with diffusion-weighted imaging (DWI) and MRS sequences was also performed. Due to the presence of equivocal MRI findings, subsequent diagnostic assessment included a comprehensive laboratory examination to rule out infectious agents, rheumatologic disorders, toxic and drug-induced encephalopathy, paraneoplastic neurological syndromes (PNSs), and possible AE; analyses were carried out both on serum and cerebrospinal fluid (CSF). The most common agents responsible for infectious encephalitis (i.e., HSV, VZV, EBV, CMV, enterovirus, HHV) were tested by polymerase chain reaction on CSF. Similarly, autoantibodies directed towards neural surface antigens (i.e., NMDAR, LGI1, CASPR2, GABABR, AMPAR) and onco-neuronal autoantibodies (i.e., HU, CV2/CRMP5, PNMA2, RI, YO) were also screened; circulating autoantibody profiling of the most common systemic autoimmune disorders was also performed (i.e., anti-TPO, anti-tTG, APL, ANA, anti-CTD, anti-CCP, anti-dsDNA, anti-SSa, and anti-SCL). After MRI examination, patients were finally tested for excluding the presence of an underlying extra-cranial tumor resorting to whole-body 18F-fluorodeoxyglucose positron emission tomography/computerized tomography (WB 18F-FDG PET/CT).

All the patients underwent comprehensive neurological examination and MRI follow-up, showing the presence of a single intracranial lesion with contrast-enhancement and mass effect; patients were thus referred to neurosurgery for brain biopsy and partial or gross total tumor resection (depending on lesion localization and dimension). Final GBM diagnosis was confirmed at pathological examination.

## 3. Results

A total of five patients were identified. All patients were female, with a median age at symptom onset of 57.4 ± 10.3 years; previous oncological anamnesis was negative in 4/5 cases, whereas one single patient had a past medical history of colorectal adenocarcinoma and basalioma in stable remission in the last 10 years. Concerning previous autoimmune disorders, 2/5 patients were reported with chronic thyroid disorders under medical treatment, of which one also reported repeated miscarriage in reproductive age (no further investigations).

**Figure 1:**
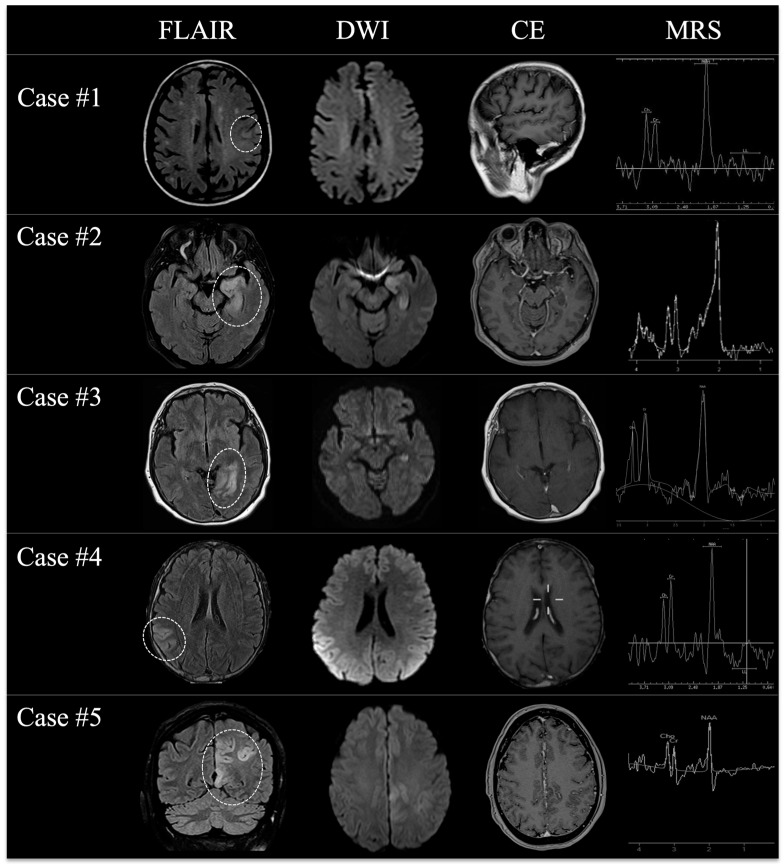
A panel showing the most relevant MRI changes (*dotted lines*) of all patients at the first time-point MRI examination on FLAIR (first column), DWI (second column), contrast-enhanced T1w (third column), and MRS (fourth column) once the initial suspicion of AE was raised.

**Figure 2:**
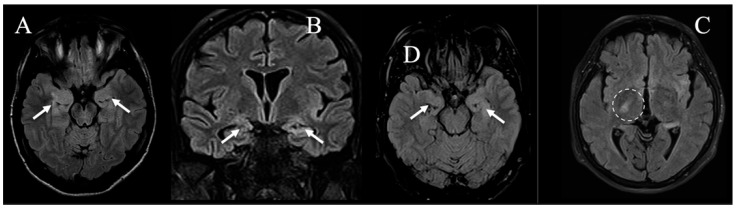
The most relevant FLAIR images at the first time-point MRI examination (**A**–**C**) in patients with bilateral mesial-temporal signal abnormalities associated with the distant primary alteration (respectively, cases #1, #4, and #5, *white arrows*), and (**D**) in a single patient with subtle changes in the internal capsule contralateral to the distant primary alteration (namely case #2, *dashed line*).

All patients were referred to the emergency department with acute–subacute cognitive deterioration and suspected focal seizures (5/5), with one case rapidly evolving in generalized convulsive status epilepticus requiring transitory admission to the intensive care unit with prompt seizure resolution. Clinical examination confirmed cognitive and behavioral changes in all subjects, coupled to variable memory deficit and temporo-spatial disorientation; in 1/5 cases, transient focal neurological deficit (speech impairment) was also observed, whereas in 1/5 cases, low-grade fever was documented at admission. An unenhanced emergency CT scan was reported negative, while EEG showed epileptiform discharges consistent with seizure semiology in all the cases. Subsequent MRI examination revealed the presence of cortical T2w hyperintensities with feeble/no DWI restriction and no enhancement after contrast media injection; all main lesions were asymmetrical and unilateral; 3/5 cases had bilateral mesial-temporal signal abnormalities associated with the distant primary alteration; 1/5 cases had subtle changes in the internal capsule contralateral to the primary alteration; in 3/5 cases, main alterations were located in the extra-limbic system, while in 2/5 cases, the limbic system was involved (mesial or posterior temporal lobe/para-hippocampal region). Regarding advanced MRI techniques, DSC PWI was unrevealing, while MRS showed mild NAA reduction in 1/5 patients and partial Cho/Cr ratio inversion in 2/5 patients. A panel showing the most relevant findings concerning main lesions at the first time-point MRI in all patients is shown in Figure 1, while signal abnormalities associated with the distant primary alteration are shown in Figure 2. DTI and fMRI were not performed in any subject. In addition, in all cases, WB 18F-FDG PET/CT excluded the presence of unrecognized underlying primary/recurrent extra-cranial tumor. CSF analysis was substantially unremarkable or unspecific in all subjects (oligoclonal band positivity in 1/5 cases and borderline rise in protein levels in 1/5 cases), whereas serum analyses revealed anti-CRMP5, anti-HU, and ANA-positivity in 1/5 cases, isolated ANA positivity in 2/5 cases, anti-NMDAR positivity in 1/5 cases, and anti-TPO and APL positivity in 1/5 cases. Considering the above-mentioned data collection, the suspicion of possible AE was then raised, and patients experienced the same empiric treatment based on a combination of high-dose intravenous methylprednisolone, antiseizure medications (different in each case), and standard coverage for viral/bacterial CNS infections (then discontinued due to negative CSF examinations); a table showing the application of the 2016 diagnostic criteria for possible autoimmune encephalitis by Graus et al. in the proposed case series is provided as Supplementary Materials. However, in order to manage diagnostic uncertainty related to the diagnosis of possible AE and, due to the presence of somehow unconvincing MRI findings, a close clinical–radiological longitudinal follow-up was always planned. A summary of demographical, clinical, laboratory, and imaging findings of all patients at symptom onset is shown in Table 1.

**Figure 3:**
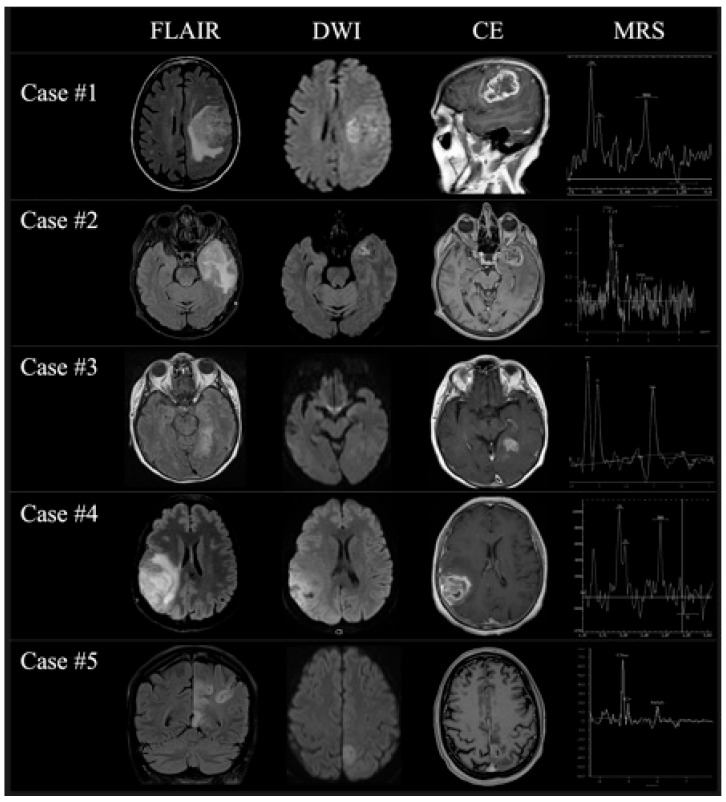
A panel showing the most relevant MRI changes of all patients at the second time-point MRI examination on FLAIR (first column), DWI (second column), contrast-enhanced T1w (third column), and MRS (fourth column) once the diagnosis of GBM was finally considered.

Clinic-radiological follow-up was performed between 1 and 3 months from the first MRI examination, depending on the response to treatment and symptom relapse or deterioration; in 1/5 patient, the response to medical therapy was complete and sustained; in 2/5 patients, the response was partial with the persistence of milder symptoms, whereas in the remaining 2/5 cases, relapse or worsening of the pre-existing symptoms was gradually observed. In all cases, longitudinal MRI showed the presence of a single large irregular intra-axial lesion with ring enhancement and central necrosis, coupled to infiltrative edema of the surrounding brain tissue; DWI and MRS suggested increased cellular density and turnover. It should be noted that all lesions, despite the increase in size and the signal changes, grossly co-localized with the main cortical alterations visible at the first MRI scans; a panel showing the most relevant findings at the first second-point MRI in all patients is shown in Figure 3. Gross total tumor resection was possible in 2/5 cases, partial resection in 2/5 cases, while in one case, biopsy was proposed; pathological examination confirmed the imaging suspicion of high-grade glioma (ABB. HGG) and wild-type isocitrate dehydrogenase (ABB. IDH) (4/5 unmethylated and 1/5 methylated). A summary of clinical, pathological, and imaging findings of all patients at the time of follow-up MRI is shown in Table 2.

Despite the subsequent prompt intensive multimodal treatment based on a combined surgical approach, radiotherapy and chemotherapy, the global outcome of these patients was poor, with an overall survival (ABB. OS) of 10 ± 4 months from GBM diagnosis.

## 4. Discussion

Encephalitis is a severe inflammatory disorder of the brain recognizing a wide spectrum of possible causes, mainly encompassing infectious disorders (especially viral) and immune-mediated reactions spreading to the CNS. Among immune-mediated disorders, AE represents one of the most complex diagnoses, requiring the preliminary exclusion of other unrecognized medical conditions responsible for overlapping signs and symptoms, and the ex juvantibus confirmation according to the most recent existing criteria [13,14,15,16]. The suspected AE diagnosis generally requires the identification of autoantibodies against the cell-surface, synaptic, or intracellular antigens [17,18]; however, at present, univocal causative antibodies have not yet been recognized [14,19,20]. AE diagnostic assessment is further complicated in the case of antibody-negative patients (accounting for about 7–10% of all AE cases), in which differential diagnosis largely relies on the exclusion of other causes and on a prompt treatment response [21,22]. Similarly, autoantibody positivity does not always imply AE, with some antibodies also being present in different clinical scenarios such as neuroinflammatory or neurodegenerative disorders [23,24,25,26,27]. As autoantibody testing is not always available in the daily clinical routine and as autoantibody-negativity/positivity is supportive but not strictly decisive for AE exclusion/confirmation, antibody status has not been included among the required AE diagnostic criteria in the most recent consensus paper on the topic [16]. Therefore, a crucial passage in possible AE diagnosis relies on the reasonable exclusion of alternative causes that may somehow mimic an initial AE presentation, especially in the case of atypical or unspecific clinical onset. MRI findings, associated with clinical and laboratory findings, are probably the weapon of major impact in distinguishing AE from possible mimics; in this light, being familiar with imaging and the clinical features of AE will allow for the most correct diagnosis or the most appropriate patient management (in-depth diagnostic exploration, strict instrumental follow-up, brain biopsy, and so on) [28,29]. Among mimics, intracranial malignancies (including HGG) represent one of the most insidious differential diagnoses, with important prognostic implications in the case of diagnostic and subsequent therapeutic delay [20].

In line with our retrospective observations, recent findings suggested how GBM at early onset may occasionally bear resemblance to various forms of encephalitis, including autoimmune, viral, or paraneoplastic [28,29,30]. Indeed, it is already known how HGG at diagnosis can occasionally be mistaken for viral encephalitis or for other inflammatory and demyelinating disorders [31,32,33,34,35,36,37], while the differential diagnosis with AE at present seems to be more challenging and controversial. In this latter regard, two recent comprehensive studies reviewed patients initially misdiagnosed with AE, showing how a significant subset (ranging between 2 and 9.5% cases) was later unexpectedly diagnosed with gliomas, including GBM [9,10]; several isolated reports also described similar AE presentations for GBM, sometimes associated with antibody positivity such as voltage-gated potassium channel complex [8,38,39], sometimes serologically negative [40,41]. This emerging evidence underscores the need for a thorough diagnostic approach, incorporating advanced neuroimaging, CSF analysis, and serological testing to distinguish between these conditions and ensure appropriate clinical management. Based on our experience, AE-like GBM presentation is an uncommon yet complex condition with significant therapeutic and prognostic consequences. The reported cases highlight the necessity of a prudent diagnostic approach combined with a thorough and continuous instrumental follow-up in patients with suspected AE and atypical MRI findings. The onset of acute–subacute symptoms, such as memory impairment, behavioral disturbances, and seizures in otherwise healthy individuals, should serve as an initial warning sign, emphasizing the importance of multimodal instrumental assessment. When an initial CT scan yields inconclusive results, a contrast-enhanced MRI becomes essential to exclude active CNS lesions. Key radiological indicators suggestive of HGG include blood–brain barrier disruption, irregular contrast enhancement, varying degrees of central necrosis or hemorrhage, and diffuse infiltration of adjacent tissues [42]. However, in cases with subtler abnormalities and no evident solid contrast enhancement, diagnosing GBM may be particularly difficult, especially when EEG, CSF analysis, or autoimmune serology provide ambiguous or non-specific results. In such rare and uncertain scenarios, while the hypothesis of AE is reasonable but not definitely confirmed, close imaging follow-up remains crucial to rule out alternative diagnoses, and brain biopsy should be considered when needed. In our cases, the clinical decision always favored strict MRI follow-up within 4 to 8 weeks, depending on the patient’s response to initial treatments. This modest delay, attributable to the initial diagnostic uncertainty caused by an atypical clinical and radiological presentation, likely did not have a significant impact on the final prognosis, which was largely determined by the biological aggressiveness of the primary lesions and the feasibility of achieving a radical surgical resection (indeed, it is well known that gross total tumor resection is one of the most critical determinants of survival in patients with HGG, as maximal tumor removal is consistently associated with improved overall outcomes and survival rates). In light of these considerations, atypical MRI findings (also coupled to suboptimal response to therapy) is probably the most important clue for reconsidering AE diagnosis and options for strict imaging follow-up. The theory given most credit today is that GBM can elicit an immune response able to trigger clinical symptoms, even before the cancer reaches a radiologically detectable size. This phenomenon may occur due to the release of tumor-associated antigens that provoke an immune reaction, leading to neuroinflammation and autoimmune-like manifestations [12,43,44]; this is supported by reported associations of GBM with PNS and AE, suggesting that tumor-induced immune dysregulation can manifest when the tumor is at the limits of MRI detection. In such exceptional cases, conventional MRI techniques may not reveal an obvious mass, emphasizing the need for deeper approaches, including multimodal imaging (i.e., advanced MRI techniques, nuclear medicine), CSF analysis, and serological testing, as well as early biopsy in limited cases. Understanding the interplay between AE and GBM is essential to optimize patient care, avoiding misdiagnosis while ensuring timely intervention for both conditions. However, at present, the interaction between GBA and AE is still poorly understood [43,45,46]; in some cases, GBM has been described as a possible trigger for paraneoplastic immune response, resulting in AE-like presentations; in some other cases, GBM has been described as an AE mimic, with patients exhibiting symptoms like memory disturbances, behavioral changes, and seizures, alongside atypical MRI findings and the absence of CSF/serum criteria for AE diagnosis; finally, in rare instances, GBM and AE have even been identified as coexisting conditions. Therefore, based on the available knowledge, it is not yet possible to determine whether this represents a mimic, an association, a casual coexistence (double trouble), or a variable combination of these three options.

A final noteworthy remark applies to the findings in our patient series. Despite the fact that it is well-known that neurological antibody-mediated disorders have a mostly female predominance [47], it is currently not possible to determine whether a similar epidemiological pattern can also apply to the rare cases of AE-like GBM presentation; in our case series, middle-aged female patients appear to be the most affected group (irrespective of their medical history or existing comorbidities); however, given the relatively small number of collected cases, these findings cannot be considered generalizable. This is all the more true when considering there is no evidence in favor of an increased risk of brain tumors in patients with autoimmune disease [48,49].

The main limitations in our patients’ series are represented by the retrospective design of this study and the relatively low number of collected cases. Despite this retrospective analysis allowing for a clear depiction of the temporal sequence between symptom onset and final diagnosis, records and data were not specifically designed for the study; therefore, it is not always homogeneous and quantitatively comparable. As patients are referred to different specialized or non-academic medical centers, possible confounding factors were not always explored in the same way and with the same timing, and no PWI analysis was homogeneously performed at the first MRI examination. Moreover, despite all our patients having underwent WB 18F-FDG PET/CT for excluding the presence of an underlying extra-cranial tumor, no specific nuclear medicine modality for brain tumor detection or characterization was applied; it is general knowledge that techniques such as brain SPECT and PET with specific radiotracers can help in differentiating the tumor from potential non-neoplastic mimics [50]; however, in our series, the possible impact of these techniques on the diagnostic flow-chart has not been explored. Finally, potential losses both to the first diagnosis and to follow-up may have underestimated the number of patients finally enrolled in the analysis.

Concerning possible connections between glial neoplasm and immune-mediated neurologic phenomena, the speculation on their association/correlation is still little-known and no systemic data have been collected in the scientific literature at present. Recent studies suggest an emerging role for artificial intelligence techniques applied to neuroimaging in distinguishing GBM-mimicking encephalitis from AE [51,52], as well as for seroreactivity profiling in glioma autoantibody signature identification and minimally invasive cancer detection [53,54]; these observations, despite being very embryonic, provide a useful basis for reflecting on future solutions in the case of GBM encephalitis-like presentation.

## 5. Conclusions and Future Directions

HGG onset might rarely begin with features observed in AE, just as it is true that the presence of autoantibodies against various neuronal cell antigens might be observed either without or in association with cancer. In this light, neuroimaging may play a crucial role in depicting different scenarios, allowing for differential diagnosis among different conditions. However, despite diagnostic efforts, it has become increasingly evident that rare, atypical GBM presentation may still incorrectly lead to limbic or AE diagnosis. In our case series, we collected data exclusively from female patients diagnosed with GBM who initially presented with signs and symptoms indicative of AE. Early MRI evaluations showed non-specific alterations of brain parenchyma without definitive radiological features of neoplasia, and CSF analysis was largely unremarkable although some patients did show the presence of autoantibodies. Our experience demonstrated how, in selected cases of considerable diagnostic uncertainty where diagnostic criteria for definite AE are not fulfilled, close MRI follow-up is essential, as it is one of the most reliable tools for confirming the diagnosis of possible AE; in the case of extreme and persistent incertitude, brain biopsy can also be envisaged to minimize the margin of error and avoid diagnostic pitfalls.

## Figures and Tables

**Table 1:** Demographical, clinical, laboratory, and imaging findings of all patients at symptom onset.

	Case #1	Case #2	Case #3	Case #4	Case #5
**Age**	50	65	70	57	45
**Sex**	F	F	F	F	F
**Clinical presentation at admission**	Seizures, memory deficit, temporo-spatial desorientation, mild right upper limb motor deficit	Cognitive decline, memory deficit, low-grade fever, seizures	Progressive bizzarre behavioral changes, temporo-spatial disorientation, focal seizures	Psychomotor agitation, speech impairment lasting a few days, left arm clonic movements	Bizzarre behavioral changes, right arm tonico-clonic movements
**CT**	NSTR	NSTR	NSTR	NSTR	NSTR
**EEG**	Left epileptiforme discharges	Left epileptiform discharges	Left frontotemporal epileptiform discharges	Right hemispheric slow periodic discharges	Left hemispheric slow periodic discharges
**Major MRI findings**	Left parieto-occipital and thalamic FLAIR hyperintensities, with bilateral mesial temporal changes	Left mesial temporal lobe cortical thickening with FLAIR hyperintensity (subtle changes in right internal capsule)	Posterior left mesial temporal lobe FLAIR hyperintensity and ribbon-like cortical thickening	Right cortical frontal precentral FLAIR hyperintensity with no cortical thickening; subtle bilateral temporal changes (left > right)	Left cortical frontal-parietal, precentral FLAIR hyperintensity with ribbon-like thickening; subtle bilateral temporal changes (left > right)
**Symmetry**	Asymmetric; main alteration unilateral	Asymmetric; main alteration unilateral	Asymmetric; main alteration unilateral	Asymmetric; main alteration unilateral	Asymmetric; main alteration unilateral
**Localization**	Extra-limbic	Limbic system	Limbic system	Extra-limbic	Extra-limbic
**CE**	Absent	Absent	Absent	Absent	Absent
**MRS**	Partial Cho/Cr ratio inversion	NSTR	Moderate NAA reduction	NSTR	Partial Cho/Cr ratio inversion
**CSF**	NSTR	NSTR	Anti-HSV 1/2 IgG	OCB	Borderline rise in protein levels (0.45 g/L)
**WB PET/CT**	NSTR	NSTR	NSTR	NSTR	NSTR
**Autoimmune serology**	ANA	ANA	Anti-CRMP5, anti-HU, and ANA	Anti-NMDAR	Anti-TPO and APL
**Oncologic history**	NSTR	NSTR	History of colo-rectal cancer and basalioma (stable remission)	NSTR	NSTR
**Previous autoimmune disorders**	NSTR	NSTR	NSTR	Hashimoto thyroiditis, repeated miscarriage	Chronic thyroid disease
**Suspected AE diagnosis**	Possible autoimmune encephalitis (Ab-neg)	Possible autoimmune encephalitis (Ab-neg)	Possible paraneoplastic vs. autoimmune encephalitis	Possible anti-NMDA receptor encephalitis	Possible encephalopathy associated with autoimmune thyroid diseases

Legend (alphabetical order): AE = autoimmune encephalitis; ANA = antinuclear antibodies; APL = antiphospholipid; CE = contrast enhancement; Cho/Cr ratio = choline/creatine ratio; CRMP5 = collapsin response-mediator protein-5; CSF = cerebrospinal fluid; CT = computed tomography; EEG = electroencephalogram; FLAIR = fluid attenuated inversion recovery; HSV = herpes simplex virus; HU = anti-neuron-specific cell nuclear antibodies; MRI = magnetic resonance imaging; MRS = magnetic resonance spectroscopy; NAA = N-acetylaspartate; NMDAR = N-methyl-D-aspartate receptors; NSTR = nothing significant to report; OCB = oligoclonal bands; TPO = Thyroid peroxidase; WB PET/CT = whole-body positron emission tomography/computed tomography.

**Table 2:** Clinical, imaging, pathological, and survival data of all patients at the time of follow-up MRI.

	Case #1	Case #2	Case #3	Case #4	Case #5
**First to second MRI scan interval (weeks)**	4	6	8	4	6
**Response to therapy**	Partial	Partial	Sustained	Poor	Poor
**Clinical presentation at follow-up**	Persisting mild right upper limb motor deficit	Persisting cognitive and behavioral deterioration	NSTR	Relapsing speech disorder and left arm clonic movements	Behavioral changes and altered consciousness
**Major MRI findings**	Expansile edematous intra-axial lesion	Expansile edematous intra-axial lesion	Expansile edematous intra-axial lesion	Expansile edematous intra-axial lesion	Expansile edematous intra-axial lesion
**Localization**	Left parieto-occipital lobe	Left temporal lobe	Left temporal lobe	Right fronto-parietal region	Frontal lobe
**CE**	Irregular ring enhancement with inner necrotic core	Irregular ring enhancement with inner necrotic core	Irregular patchy enhancement	Irregular ring enhancement with inner necrotic core	Irregular ring enhancement with inner necrotic core
**MRS**	Increased Cho/NAA ratio, inverted Cho/Cr ratio, mild Lip-Lactate peak	Artifacts (significant NAA depletion, inverted Cho/Cr ratio)	Increased Cho/NAA ratio, inverted Cho/Cr ratio	Increased Cho/NAA ratio, inverted Cho/Cr ratio	Increased Cho/NAA ratio, inverted Cho/Cr ratio
**Surgery**	Biopsy	Gross total resection	Gross total resection	Partial resection	Partial resection
**Pathology**	GBM IDH wild-type, unmethylated	GBM IDH wild-type, unmethylated	GBM IDH wild-type, methylated	GBM IDH wild-type, unmethylated	GBM IDH wild-type, unmethylated
**OS (months)**	7	18	15	9	7

Legend (alphabetical order): CE = contrast enhancement; Cho/Cr ratio = choline/creatine ratio; MRI = magnetic resonance imaging; MRS = magnetic resonance spectroscopy; NAA = N-acetylaspartate; NSTR = nothing significant to report; OS = overall survival (measured from first MRI examination).

## Data Availability

Data supporting findings of this study are available from the first author upon reasonable request, due to privacy concerns.

## Supplementary Materials

The following supporting information can be downloaded at https://www.mdpi.com/article/10.3390/jcm14113807/s1. Table S1. Table showing the application of the 2016 diagnostic criteria for possible autoimmune encephalitis by Graus et al. in the proposed case series [16].

## Abbreviations

AE	autoimmune encephalitis
CNS	central nervous system
CSF	cerebrospinal fluid
CT	computed tomography
DCE	dynamic contrast enhancement
DSC	dynamic susceptibility contrast
DTI	diffusion tensor imaging
DWI	diffusion-weighted imaging
EEG	electroencephalography
fMRI	functional magnetic resonance imaging
GBM	glioblastoma
MRI	magnetic resonance imaging
MRS	magnetic resonance spectroscopy
PNS	paraneoplastic neurological syndromes
PWI	perfusion weighted imaging
WB 18F-FDG PET/CT	whole-body 18F-fluorodeoxyglucose positron emission tomography/computerized tomography
