# Supplementary material for: Autoimmune Encephalitis-like Presentation of Glioblastoma: Get to Know This Rare Occurrence
Source: J Clin Med. 2025 May 29;14(11):3807. doi: 10.3390/jcm14113807 (PMC12155702; doi:10.3390/jcm14113807)
Supplement: Supplementary file 1 [file jcm-14-03807-s001.zip › jcm-3587152-supplementary.pdf]

Supplementary materials

Table S1. A table showing the application of the 2016 diagnostic criteria for possible autoimmune encephalitis by Graus et al. in the proposed case series.

**Diagnostic criteria for possible AE, according to Graus et al. Lancet Neurol. 2016 Apr.15(4)**		**Case #1**	**Case #2**	**Case #3**	**Case #4**	**Case #5**
1 Subacute onset (< 3months) of working memory deficits,altered mental status or psychiatric symptoms		**present**	**present**	**present**	**present**	**present**
2 At least ONE of the following		**present**	**present**	**present**	**present**	**present**
	• New focal CNS findings	✗	✗	✗	✓	✗
	• Unexplained seizures	✓	✓	✓	✓	✓
	• CSF pleocytosis (>5cells per mm^3)	✗	✗	✗	✓	✓
	• MRI compatible with encephalitis (*)	✓	✓	✓	✗	✗
3 Reasonable exclusion of alternative causes		**present**	**present**	**present**	**present**	**present**
	• HSV	✗	✗	✗	✗	✗
	• HHV	✗	✗	✗	✗	✗
	• HIV	✗	✗	✗	✗	✗
	• Whipple	✗	✗	✗	✗	✗
	• Neurosyphilis	✗	✗	✗	✗	✗
	• Status epilepticus	✗	✗	✗	✗	✗
	• HGG (**)	✗	✗	✗	✗	✗

Legend (alphabetical order): AE = autoimmune encephalitis; CSN = central nervous system; CSF = cerebrospinal fluid; HGG = high-grade glioma; HHV = human herpesvirus; HIV = human immunodeficiency virus; HSV = herpes simplex virus; MRI = magnetic resonance imaging; (*) hyperintense signal on T2w FLAIR restricted to one or both medial temporal lobes, or in multifocal areas involving grey matter, white matter, or both; (**) no typical MRI findings at first MRI examination, and no contrast-enhancement.
